# Midwifery

**Published:** 1837-07

**Authors:** 


### MIDWIFERY.

### *Fracture of the Skull and Rupture of the longitudinal Sinus during Natural Labour.* By Dr. MICHAELIS, of Kiel.

A WOMAN, aged thirty-six, was taken in labour with her first child on the 27th February, 1836. After slight pains for thirty-six hours, the waters came away at four P. M. of the 29th. At this time the midwife found the os uteri open, but full and hard. At eight o'clock on the following morning, Dr. M. was called, and found the patient, as is common in Germany, on the labour-chair, with sufficient but not severe labour pains, the vagina extremely tender, dry and hot, and the perineum tense, and the child's head completely in the pelvis. The woman was put into bed, and warm cataplasms applied to the genitals; and in two hours things were completely changed, the parts being now only moderately swelled, lubricated, and not painful, while the head advanced under good pains, only apparently strongly jammed against the immoveable coccyx. At eleven o'clock the child was born. Examination immediately after the child was born detected no deformity of the pelvis, the diameter of the outlet being certainly not less than three inches and three quarters. The child breathed both during birth and immediately after, but then died.

The head was much disfigured, and, on examining it carefully, the following appearances were found: 1. The frontal bones were normal as to structure and uninjured, but so flattened that the frontal and parietal portions lay nearly in the same place. 2. The fontanelle and anterior two-thirds of the sagittal suture projected high up, and the sagittal borders of the parietal bones were thrown open and partook of the displacement. 3. The left parietal bone was not otherwise affected, and, with the exception of a few points which were very thin, it was well-formed. 4. In the posterior third part of the sagittal suture, where the parietal bones were firm and well formed, and the suture only two lines in width, were seen small livid portions of the longitudinal sinus forced between the bones. 5. The occipital bone was flattened and forced deep under the parietal bones, but not otherwise injured. 6. The right parietal bone, which during birth had been directed towards the promontory of the sacrum, was found covered anteriorly and above with effused blood, and, on removal of the periosteum, was found fractured in five places. These fractures or fissures were as follows: *a.* a small fissure near the *tuber ossis frontis,* four lines long; *b.* a much larger one running from the fontanelle through the centre of the parietal bone, very nearly two inches in length; *c. d.* two small fissures half an inch in length, near the sagittal suture. There were also four small openings, from incomplete ossification, in the same bone, which was throughout very thin.

On opening the skull, there was found no extravasation beneath the fissures, but posteriorly under the sagittal suture the longitudinal sinus was found ruptured, and there was an extensive coagulum on the cerebrum on both sides, under the dura mater, and on the tentorium cerebelli.

The peculiarity of this case is, the occurrence of so extensive injury during natural labour and with a well-formed pelvis; and is explained by the natural weakness of the bone and the unfavorable position of the head during birth.

*Neue Zeitschrift für Geburtskunde.* B. iv. H. 3. 1836.

